# Clinical characteristics and quality of life in children with PFAPA syndrome and Behçet’s disease

**DOI:** 10.1093/rap/rkaf015

**Published:** 2025-02-11

**Authors:** Asli Gürel Bedir, Sara Sebnem Kilic

**Affiliations:** Department of Pediatric Immunology and Rheumatology, Bursa Uludag University, Bursa, Turkey; Department of Pediatric Immunology and Rheumatology, Bursa Uludag University, Bursa, Turkey

**Keywords:** Behçet’s disease, PedsQL questionnaire form, PFAPA, HLA-B

## Abstract

**Objective:**

Periodic fever, aphthous stomatitis, pharyngitis, cervical adenitis (PFAPA) syndrome and Behçet’s disease (BD) are non-monogenic autoinflammatory disorders with common clinical characteristics and genetic features. We aimed to review both patients’ clinical characteristics and quality of life.

**Methods:**

Retrospective data were collected on patients’ clinical and laboratory characteristics with PFAPA and BD between 2019 and 2022. The quality of life questionnaire (Pediatric Quality of Life Inventory) form was completed by the parents of all patients and the control group.

**Results:**

The study included 150 cases aged between 2 and 18, including 60 with PFAPA, 30 BD patients and 60 healthy volunteers. The duration of attacks in males with PFAPA was longer than that in females (*P* = 0.015). During attacks, the mean acute phase reactants of PFAPA patients were higher than those of BD (*P* = 0.010). In addition, there was a statistically significant relationship between the use of colchicine and a decrease in attack frequency in BD patients at 89.29% (*P* = 0.001 < 0.05) and in PFAPA patients at 88% (*P* = 0.001 < 0.05). Precisely, 61.67% of PFAPA (*n* = 37) and 73.33% of BD patients (*n* = 22) exhibited poor quality of life regarding daily activities and school attendance.

**Conclusion:**

Limited data on the quality of life of paediatric BD and PFAPA syndrome are available. During the attacks of patients with PFAPA and BD, acute-phase reactants were higher in PFAPA patients. Colchicine is an effective medication in reducing attacks in both groups. Effective and timely treatment is indispensable to improve quality of life.

Key messages

- Fever and oral aphthae were the major symptoms of the attacks in PFAPA and BD.
- The use of colchicine reduced the attack frequency in both patient groups.
- The total score of the PedsQL form scale was lower in patient groups than in controls.

## Introduction 

Periodic fever, aphthous stomatitis, pharyngitis, adenitis (PFAPA) syndrome was first described as Marshall syndrome in 12 paediatric patients presenting with periodic fever, aphthous stomatitis, pharyngitis and cervical lymphadenopathy by Marshall and colleagues in 1987 [1]. Later, this condition was named PFAPA syndrome in 1989. Although viral and autoimmune mechanisms have been proposed in the aetiology, the exact cause is still unknown [2]. Dysregulation of cytokine release is suspected in the pathogenesis of the disease, and it has been defined as a non-inherited autoinflammatory disorder with a central role for IL-1β [3, 4]. PFAPA syndrome is characterized by recurrent fever attacks lasting 3–6 days, occurring every 3–8 weeks, typically presenting before age 5, and responding dramatically to corticosteroids [5]. While fever is present in every episode, the other three cardinal features of the syndrome, pharyngitis, aphthous stomatitis and cervical lymphadenopathy, may not be present in the same attack. Cervical lymphadenopathy (88%) is the most commonly seen finding apart from fever, followed by pharyngitis (72%) and aphthous stomatitis (70%) in PFAPA [6].

Behçet’s disease (BD) is an autoinflammatory disorder described by Dr Hulusi Behcet in 1937 and relatively common in Turkey. It is characterized by mucocutaneous manifestations, including recurrent oral and genital ulcerations, ocular manifestations (chronic uveitis) and systemic vasculitis involving arteries and veins of all sizes. The International Classification Criteria, which includes the paediatric BD criteria established by a new consensus in 2015, are used in diagnosing BD [7, 8]. Pathergy testing is not included, and oral ulcers are not mandatory for the diagnosis [8]. The main principle in treating BD is to control symptoms, reduce inflammation and prevent secondary organ damage [7].

The Pediatric Quality of Life Inventory (The PedsQL) is a quality-of-life scale that measures functional health and well-being from the patient’s point of view through questions. Healthiness is defined as physical health, emotional and social functioning by the World Health Organization [9, 10].

PFAPA and BD are considered autoinflammatory disorders, primarily driven by innate immune system abnormalities rather than autoimmune mechanisms. They are characterized by episodes of inflammation that flare up periodically and involve immune system dysfunction, though the exact mechanisms differ. In PFAPA syndrome and BD, the episodes involve fever and mouth ulcers; however, inflammation of the throat and lymph nodes in PFAPA, oral and genital ulcers, eye inflammation and skin lesions were observed in BD.

The commonality between PFAPA syndrome and BD lies in the episodic nature of their inflammatory episodes and the presence of oral ulcers.

Although there have been previous claims that PFAPA syndrome is an early stage on the path to BD, this has not been demonstrated in BD patients in our country. Furthermore, no study worldwide has compared the quality-of-life scores for these two diseases.

Our aim is to investigate whether PFAPA is an early indicator of BD in our patients and to compare the clinical and laboratory findings and the quality-of-life scores for both conditions. Additionally, we seek to identify factors that improve the quality of life and help control the diseases in both patient groups and compare these findings with healthy control children using the PedsQL scale.

## Methods

The study included 150 participants aged 2–18, including 60 with a previous diagnosis of PFAPA, 30 with BD and 60 healthy volunteers from the Department of Pediatric Immunology-Rheumatology at Bursa Uludağ University Faculty of Medicine. Study reporting was done by the STROBE guidelines [11]. Data from the patients were retrospectively reviewed.

The diagnosis of the PFAPA syndrome was established according to modified Marshall’s criteria [5]. The International Criteria for Pediatric Behçet’s Disease (PEDBD) was employed to assess the diagnosis of BD [8]. Exclusion criteria were: (1) refusal to participate, (2) positive genetic test for autoinflammatory disease and (3) age over 18 years.

Electronic files of patients meeting the diagnostic criteria for PFAPA and paediatric BD were retrospectively reviewed by a paediatric rheumatologist. Demographic data and laboratory values, including absolute neutrophil count (ANC), CRP, fibrinogen, serum amyloid A (SAA) and procalcitonin levels, were evaluated in 90 patients with PFAPA or BD during the active phase of the disease. The PedsQL form was completed with the parents of all patients with PFAPA or BD diagnosis and the healthy control group aged 2–18.

The study protocol was approved by the clinical research and ethics committee of Uludag University (ethics approval number 2022–2/18). Each participant and parent signed an informed consent form in accordance with the Declaration of Helsinki.

### Statistical analysis

Data were analysed using SPSS (Statistical Package for Social Sciences) 25 software. Categorical variables were summarized using frequency and percentages, while quantitative variables were summarized using mean and standard deviation. The normal distribution assumption was checked using the Kolmogorov–Smirnov test. When parametric test conditions were obtained, two independent group means were tested using the *t*-test, and multiple group means were tested using analysis of variance (ANOVA). When parametric test conditions were not met, two independent group means were tested using the Mann–Whitney U test, and multiple group means were tested using the Kruskal–Wallis H test. The relationship between two categorical variables was examined using the chi-square test, and the relationship between variables was examined using Pearson’s correlation coefficient if the normal distribution assumption was met and Spearman’s rank correlation coefficient if it was not met. Receiver operating characteristic (ROC) analysis was used to determine the predictive value of poor quality of life in the diagnostic groups. The significance level for hypothesis testing was set at 0.05.

## Results

The average age of diagnosis was 29 months in the PFAPA group and 114 months in the BD group. The average age of diagnosis in the BD group was higher than in the PFAPA (*P* < 0.001). According to the report, 3.33% of BD patients, 6.67% of PFAPA patients and 6.67% of patients in the control group had a weight percentile value of <3p. In comparison, 13.33% of BD patients, 5% of PFAPA patients and 6.67% of patients in the control group had a height percentile value of <3p. The difference in mean attack duration between males and females was significant in the PFAPA group (*P* = 0.015 < 0.05), with males having a longer duration of attacks than females. The rate of lymphadenopathy in the neck region was higher in PFAPA compared with BD patients and control groups (*P* < 0.001). The frequency of fever was increased in patients with PFAPA and BD compared with the control group, followed by oral aphthae as the second most common symptom. Painful skin lesions, eye redness, uveitis, testicular pain and genital ulcers were more common in BD than in the PFAPA patients and control groups and showed significant differences (*P* < 0.001). The rate of a family history of recurrent fever attacks associated with tonsillopharyngitis was statistically higher in PFAPA patients compared with the other groups (*P* < 0.001) (Table 1). The mean values of ANC (*P* = 0.010 < 0.05), CRP (*P* < 0.001) and SAA (*P* < 0.001) in disease episodes were found to be significantly lower in BD compared with PFAPA patients, while the difference in fibrinogen (*P* = 0.056 > 0.05) and procalcitonin (*P* = 0.756 > 0.05) values was not statistically significant (Table 2). HLA-B35 positivity was higher in PFAPA patients than in BD and control groups (*P* = 0.001 < 0.05). HLA-B51 positivity showed the highest frequency in the BD group, with a rate of 35.59% (*n* = 23) among all groups. HLA-B58 positivity was higher in control group patients than in BD disease and PFAPA groups (*P* = 0.009 < 0.05).

**Table 1.** The characteristic features of the patients

	BD		PFAPA		Control group		*P*-value
	*n*	%	*n*	%	*n*	%	
Fever	15	50.00%	60	100.00%	16	26.67%	<0.001
Oral ulcers	22	73.33%	35	58.33%	0	0.00%	<0.001
Frequent URTI	14	46.67%	34	56.67%	9	15.00%	<0.001
Cervical lymphadenopathy	4	13.33%	24	40.00%	1	1.67%	<0.001
Painful skin lesions	5	16.67%	0	0.00%	0	0.00%	<0.001
Redness or tearing of the eye	15	50.00%	18	30.00%	3	5.00%	<0.001
Uveitis	8	26.67%	1	1.67%	0	0.00%	<0.001
Testicular pain	3	10.00%	1	1.67%	0	0.00%	0.027
Genital ulcers	19	63.33%	1	1.67%	0	0.00%	<0.001
Joint symptoms	25	83.33%	25	41.67%	5	8.33%	<0.001
Family history of PFAPA syndrome	0	0.00%	16	26.67%	0	0.00%	<0.001
Family history of FMF	4	13.33%	15	25.00%	5	8.33%	0.041
Family history of BD	14	46.67%	3	5.00%	2	3.33%	<0.001
Parental consanguinity	2	6.67%	9	15.00%	13	21.67%	0.181
History of tonsillectomy	6	20.00%	19	31.67%	7	11.67%	0.027
History of familial tonsillectomy	9	30.00%	18	30.00%	17	28.33%	0.976
Positive history of tonsillitis in mother	9	30.00%	22	36.67%	13	21.67%	0.196
Positive history of tonsillitis in father	11	36.67%	15	25.00%	5	8.33%	0.04

URTI: upper respiratory tract infections.

**Table 2.** Laboratory data of the patients

Variables	BD			PFAPA			*P*-value
	Minimum	Maximum	Mean±SS	Minimum	Maximum	Mean±SS	
ANC (/mm3)	472	11910	4678.33 ± 2632.75	1170	30400	7065.18 ± 4940.88	0.010
CRP (mg/dl)	0.2	125	10.33 ± 28.39	0.3	314	48.96 ± 60.29	<0.001
Fibrinogen (mg/dl)	191	617.3	331.73 ± 121.92	191	806	364.38 ± 111.17	0.056
Procalcitonin (ng/ml)	0.01	0.21	0.06 ± 0.05	0	13.70	0.53 ± 2.05	0.756
Serum amyloid A (mg/l)	0	1200	112.31 ± 280.11	4.33	5030	446.12 ± 722.23	<0.001

The rate of tonsillectomy was also higher in PFAPA and BD patients compared with the control group (*P* = 0.027 < 0.05). There was a statistically significant relationship between the use of colchicine and a decrease in attack frequency in BD patients at 89.29% (*P* = 0.001 < 0.05) and in PFAPA patients at 88% (*P* = 0.001 < 0.05) (Table 3).

**Table 3.** The use of colchicine in treatment and its effect on attacks

Diagnostic groups				Did colchicine decrease attack frequency?		*P*-value
				No	Yes	
BD	Colchicine history?	No	*n*	2	0	0.023
			%	100.00%	0.00%	
		Yes	*n*	3	25	
			%	10.71%	89.29%	
PFAPA	Colchicine history?	No	*n*	10	0	<0.001
			%	100.00%	0.00%	
		Yes	*n*	6	44	
			%	12.00%	88.00%	

**Table 4.** Parent proxy-reported and child self-reported PedsQL™ scores of paediatric patients with BD, PFAPA and control group

PedsQL		BD	PFAPA	Control group	*P*-value
Physical functioning problems	Walking more than one block	65 ± 28.31	61.25 ± 39.17	84.17 ± 25.2	0.001
	Running	61.67 ± 29.16	53.33 ± 37.24	76.67 ± 29.42	0.001
	Doing sports or exercise	61.67 ± 29.16	57.92 ± 33.66	75 ± 27.23	0.010
	Low energy level	57.5 ± 27.97	68.33 ± 23.41	76.25 ± 27.4	0.005
Emotional functioning problems	Feeling frightened	58.33 ± 31.71	66.67 ± 25.49	70.83 ± 24.43	0.170
	Feeling sad or depressed	52.5 ± 28.88	65.83 ± 23.46	71.67 ± 23.23	0.008
	Feeling angry	48.33 ± 23.61	54.58 ± 28.56	67.08 ± 25.83	0.004
	Having difficulty sleeping	69.17 ± 26	74.58 ± 27.42	79.17 ± 23.99	0.190
	Feeling worried about what will happen to oneself	65 ± 29.8	73.33 ± 27.57	75.42 ± 27.81	0.235
Social functioning problems	Difficulty getting along with peers	80.83 ± 22.44	80.83 ± 24.94	82.92 ± 22.31	0.855
	Being rejected by peers	89.17 ± 22.44	87.92 ± 22.78	88.33 ± 19.78	0.794
	Being bullied by peers	90 ± 19.25	92.08 ± 17.52	84.58 ± 25.25	0.306
	Being unable to do what peers can do	81.67 ± 23.61	84.17 ± 23	85 ± 21.69	0.793
	Falling behind peers during play or activities	79.17 ± 24.64	82.92 ± 21.83	90.42 ± 16.65	0.032
School-related problems	Inability to perform preschool activities like peers do	–	84.09 ± 18.17	–	–
	Inability to attend preschool due to not feeling well	–	67.05 ± 28.23	–	–
	Inability to attend preschool due to go to the hospital	–	61.36 ± 31.55	–	–
	Inability to concentrate in class	63.33 ± 33.95	73.03 ± 26.24	73.31 ± 30.04	0.391
	Forgetting some things	57.5 ± 31.59	67.76 ± 24.6	65.68 ± 26.63	0.354
	Falling behind in lessons	68.33 ± 33.43	77.63 ± 26.5	77.97 ± 28.27	0.418
	Inability to attend school due to not feeling well	52.5 ± 27.35	58.55 ± 31.45	80.08 ± 22.16	<0.001
	Inability to attend school due to go to the hospital	27.5 ± 24.87	47.37 ± 28.35	72.03 ± 22.78	<0.001

### PedsQL survey

The total score of the PedsQL form scale was lower in BD and PFAPA patients than in controls. According to the ROC analysis of the poor life estimation value of the patient groups, it was determined that the PedsQL form scale score was <74.60 in the PFAPA group and <72.50 in the BD group. A total of 59 patients, 37 with PFAPA and 22 with BD, were considered to have a poor quality of life due to their scores falling below these values (Table 4).

## Discussion

BD and PFAPA syndrome, which can affect children of different age groups, share similar clinical symptoms such as fever, oral ulcers and arthralgia and exhibit some similarities in their pathogenesis. They both show a disturbance in the proinflammatory cytokine network where IL-1β plays an important role [12].

The presence of aphthous ulcers and inflammation in the oropharyngeal mucosa is a fundamental feature of PFAPA and other disorders such as BD and recurrent aphthous stomatitis [13]. Cantarini *et al.* [14] suggested in their study that PFAPA syndrome and BD share similar pathogenic and clinical characteristics and that patients diagnosed with BD in adulthood may exhibit symptoms consistent with PFAPA syndrome during childhood. They showed that 24 out of 80 BD cases (30%) met the diagnostic criteria for PFAPA syndrome in childhood. Our study is consistent with the literature; oral ulcers were the most common clinical finding in both patient groups. We found a rate of 46.67% for PFAPA history in patients with BD during childhood. Additionally, we found a rate of 5% for having a family member with BD in our PFAPA patients.

PFAPA usually starts in early childhood between ages 2 and 5. Feder and Salazar [15] reported that 80% of patients received a diagnosis before age five, and the mean age of the first attack was 33 months. The onset age of BD is between 8 and 12 years. In a multicentre demographic survey by Karincaoğlu *et al.* [16], the average age of initial presentation for 83 children with BD was 12.3 years. In our study, the PFAPA patient group showed a mean age of 30.06 ± 19.15 months. In the BD group, the age range was from a minimum of 36 months to 192 months, with a mean age of 114.80 ± 42.83 months at the initial diagnosis.

There is no specific laboratory test for diagnosing PFAPA syndrome, but during fever episodes, an increase in neutrophil-dominant white blood cells and elevated acute-phase reactants can be observed [17, 18]. In our study, while the average levels of ANC, CRP and SAA during fever attacks were significantly higher in PFAPA compared with BD patients (*P* = 0.010), the difference in procalcitonin levels (*P* = 0.756) was not statistically significant. The fact that procalcitonin does not increase like other acute phase reactants during inflammation attacks defines this protein as a potentially useful marker to distinguish PFAPA syndrome from infection.

Manthiram *et al.* [13] reported that the most critical 10 HLA alleles with a dominant allele frequency of more than 5% were evaluated in a European-American cohort study, and significant risk alleles for PFAPA were identified (HLA-B15:01, HLA-C08:02 HLA-B35:02, HLA-DRB1, HLA-DQA1 and HLA-DQB1). In the same survey, HLA-B15 was identified as an important risk allele for all three diseases among the six identified alleles. In a study investigating the distribution of HLA antigens in healthy Turkish citizens by the Health Research and Application Center Tissue Typing Laboratory in Turkey, data from 1903 individuals were analysed, and the frequency of HLA class I and II alleles was calculated. The most commonly seen HLA-B alleles were identified as HLA-B35 (16.3%), HLA-B51 (15.2%) and HLA-B*44 (8.3%) [19]. In our study, only HLA-B alleles were able to be screened. HLA-B51 positivity with a rate of 35.59% (*n* = 23) was detected in BD. HLA-B35 positivity was higher in PFAPA patients compared with the Behcet and control groups. Further studies are needed to understand the role of these risk variants and HLA alleles in PFAPA.

To further discuss the role of colchicine treatment in reducing PFAPA attacks independent of steroids, we reviewed relevant studies. In a retrospective survey by Gunes *et al.* [20], which included 400 children with PFAPA syndrome, regular colchicine treatment was found to extend the interval between episodes in 85% of patients (*n* = 303), with a significant increase in the duration of episode free periods from 18.8 ± 7.9 days to 49.5 ± 17.6 days. Tasher *et al.* [21] reported that the success rate of regular colchicine treatment was 89% in patients with PFAPA. In our study, colchicine treatment was administered to 83.3% (*n* = 50) of PFAPA patients, and we found a significant response in 88% (*n* = 44) of patients in terms of reducing attack frequency (Table 3). These findings suggest that regular colchicine treatment may be beneficial in PFAPA syndrome. Further research is necessary to determine the optimal dosage and duration of treatment. In our patients with BD diagnosis, the effect of colchicine on reducing aphthous ulcers was 89.29%.

In medicine, the concept of quality of life has emerged to approach patients holistically and assess their well-being from physical, psychological and social perspectives. PFAPA syndrome, as it completely disappears in almost every patient before adulthood and does not cause organ damage, is considered a benign disease compared with other periodic fever syndromes. For this reason, studies evaluating the quality of life of patients diagnosed with PFAPA are scarce. Grimwood *et al.* [22] assessed the quality of life in PFAPA patients for the first time in 2018 (*n* = 33) compared with a control group of FMF patients (*n* = 27) and found lower quality-of-life scores in the PFAPA group.

It seems that factors such as the recurrent nature of attacks in PFAPA and BD, early age of PFAPA diagnosis, lifelong treatment for BD patients, a requirement for regular doctor visits and isolation from school and play environments can negatively impact the quality of life [22, 23]. In our study, the absence of a difference in age and gender distribution among the control group compared with PFAPA and BD patients effectively eliminated the confounding effect of age and gender on the distribution of PedsQL scores among the three groups. Additionally, we determined a prediction value for the poor quality of life by assuming that the PedsQL scores in the healthy control group would be higher than those in PFAPA and BD patients. Indeed, our study supported our hypothesis by finding lower PedsQL total scale scores in PFAPA and BD patients compared with the healthy control group and a significant positive correlation between the number of attacks and PedsQL total scale scores in both groups (*P* = 0.044). At the same time, there were significant differences between the two disease groups regarding low daily activity scores and school absenteeism compared with the control group.

The limitations of our study are that it was conducted retrospectively, and the number of patients in the two disease groups was not equal.

Our study is the first to evaluate the quality of life in PFAPA and BD patients worldwide. Physicians should not consider PFAPA as a disease with no preventive treatment options and does not impair the quality of life. Colchicine is an effective agent in preventing disease attacks. In these two diseases, the aetiologies of which have not yet been fully elucidated and are characterized by recurrent fever and inflammation attacks, high acute phase reactant levels are particularly evident in PFAPA patients.

Recurrent attacks can affect patients’ daily living activities and school performance. Children in both patient groups can be evaluated with multidimensional assessment scales, which can improve the quality of care and treatment provided to these children and determine the areas primarily affected by the disease. Although the quality-of-life scores depend on whether they are collected during the inflammatory or asymptomatic period, the main reason for the low quality-of-life scores is the continuation of regular inflammatory attacks. Further multicentre studies are needed to confirm our results and correlate quality of life with disease activity scores.

## Data Availability

Data available via Figshare: https://doi.org/10.6084/m9.figshare.26324008.v1
